# A Review of Experiential School-Based Culinary Interventions for 5–12-Year-Old Children

**DOI:** 10.3390/children8121080

**Published:** 2021-11-23

**Authors:** Annemarie E. Bennett, David Mockler, Cara Cunningham, Corina Glennon-Slattery, Charlotte Johnston Molloy

**Affiliations:** 1 Trinity Centre for Health Sciences, St James’ Healthcare Campus, D08 W9RT Dublin, Ireland; mocklerd@tcd.ie; 2 Community Nutrition and Dietetic Service, Health Service Executive, Clonbrusk, Athlone, N37 P8P8 Co Westmeath, Ireland; cara.gray@hse.ie; 3 Primary Care Network 7, Health Service Executive, Primary Care Centre, Harbour Road, Mullingar, N91 V6R9 Co Westmeath, Ireland; corina.glennon@hse.ie; 4 Community Nutrition and Dietetic Service, Health Service Executive, St Loman’s Healthcare Campus, Mullingar, N91 X36E Co Westmeath, Ireland; charlotte.johnston@hse.ie

**Keywords:** cookery, culinary intervention, children, school, primary school, elementary school

## Abstract

Cooking is an essential skill and the acquisition of cooking skills at an early age is associated with higher diet quality. This review aimed to describe the characteristics of school-based experiential culinary interventions and to determine the value of these to child (5–12 years) health outcomes. Interventions were eligible for inclusion if they took place in school during school hours, included ≥3 classes, and had a control group. Interventions published up to May 2021 were included. The databases searched were PubMed, CINAHL, and EMBASE, and the grey literature was searched for published reports. The search strategy yielded 7222 articles. After screening, five published studies remained for analysis. Four studies targeted children aged 7–11 years, and one targeted children aged 5–12 years. The interventions included food tasting, food gardening, and/or nutrition education alongside experiential cooking opportunities. Improvements were evident in self-reported attitudes toward vegetables, fruits, and cooking, and two studies reported small objective increases in vegetable intake. School-based experiential cookery interventions have the potential to positively impact health-related aspects of the relationship children develop with food. However, a greater number of long-term methodologically rigorous interventions are needed to definitively quantify the benefits of such interventions.

## 1. Introduction

It is well-known that dietary habits in childhood have short- and long-term consequences for physical [1], mental [2], and psychosocial [3] health. Primary school-aged (i.e., 5–12 years) children are identified as having suboptimal intake of fruits and vegetables and excessive intake of processed meats and refined foods [4,5,6,7,8]. Trends such as these are contributing to the persistent challenges of overweight and obesity among younger age groups [5,9,10] and lifetime risk of chronic disease [11]. 

Cooking is an essential life skill, and the ability to cook is often associated with improved diet quality [12,13]. Learning cooking skills at an early age is reported as being important for cooking confidence and practice, and for improving nutritional intake [12]. Although children consume most of their nutritional requirements at home [4,14], schools remain a key setting in which to emphasize and impart health-promoting behaviors. Schools are well-positioned to host health-related interventions, as they have the advantage of guaranteeing access to the population of interest and they often have the infrastructure needed to facilitate interventions, such as large spaces [14]. Importantly, since schools support such a diverse group of children, interventions can impact groups that are typically considered hard to reach but that stand to benefit the most from such interventions, such as socioeconomically disadvantaged groups [15]. 

This review sought to examine the content and impact of school-based culinary interventions. Only interventions implemented during standard school hours were included, as these interventions are available to all children regardless of gender [16], logistics [17], or socioeconomic status [18]. In addition, the review sought to identify the commitment required from school staff to facilitate an effective health-promoting intervention during school hours, as logistical challenges are commonly cited as barriers to the integration of health interventions into a school day [19,20,21]. As such, this review aimed to describe the characteristics of school-based culinary interventions that take place during school hours, and to determine the value of these for child health outcomes. 

## 2. Materials and Methods

This mixed-methods [22] review included interventions that aimed to teach children aged 5–12 years cookery skills, using experiential learning techniques. “Cookery skills” encompassed the skills required to prepare hot and cold meals, including washing, peeling, chopping, dicing, grating, mixing or otherwise combining, and heating food ingredients. Only interventions that took place in a school setting, during school hours, included ≥3 classes, and had a control group were eligible. Interventions that exclusively took place during school hours were included to explore the practicality of integrating cookery skills into the school curricula. A three-class minimum was specified to reflect key stages of learning, i.e., to enable children an opportunity to acquire, become proficient in, and maintain culinary skills appropriate to their stage. Control groups were specified to enable meaningful comparison of outcomes. Studies with and without parental involvement in the intervention were included. Outcomes of interest included changes in cookery skills, food-related knowledge, and/or dietary outcomes. Interventions were excluded if they involved only demonstrations (i.e., no hands-on opportunities for children to cook), were targeted at children with diagnosed food-related health conditions (e.g., coeliac disease, phenylketonuria), had <3 classes, or no control group. Studies published in a language other than English were also excluded. 

Interventions published up to 31 May 2021 were included. There was no minimum limit on the dates of the literature search. The databases searched were PubMed, CINAHL, and EMBASE. An exhaustive search was conducted using keywords and MESH terms, such as cooking, cookery, food education, nutrition education, health, school, school child, student, primary school, elementary school, instruct, teach, skill, and technique (Box 1). A medical librarian (D.M.) assisted with the search strategy. The grey literature was also searched for published reports on school-based cookery interventions.

Box 1 **Search strategy applied to EMBASE.**

‘cooking’/exp AND (‘education’/exp OR ‘nutrition education’/exp OR ‘nutritional science’/exp OR ‘feeding behavior’/exp OR ‘nutrition’/exp)
((Cookery OR cooking OR cook OR food OR feeding OR eat* OR nutrition) NEAR/3 (educat* OR instruct* OR teach* OR demo? OR demonstration* OR skill* OR technique* OR learn* OR program* OR classes)):ti,ab
#1 OR #2
(‘school child’/exp OR ‘school’/de OR ‘kindergarten’/exp OR ‘primary school’/exp)
(child* OR teenager* OR adolescen* OR kid? OR pupil* OR student* OR school*):ti,ab
#4 OR #5
#3 AND #6

**Figure 1:**
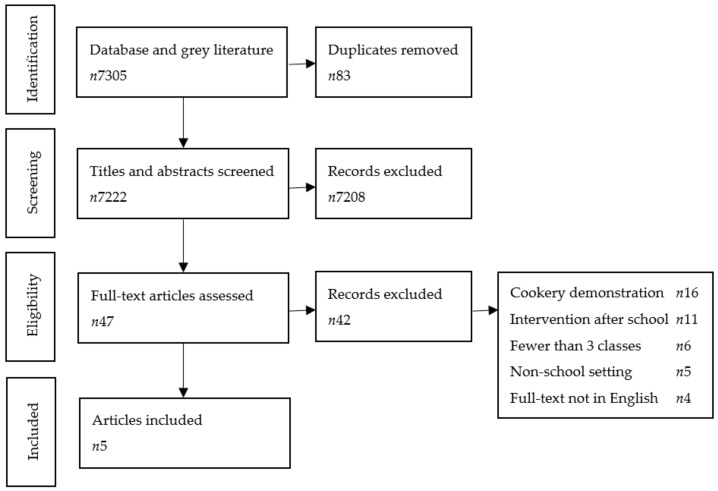
Flow chart depicting each stage of study selection.

The results of the search were imported into Endnote (Thompson Reuters Endnote X9). Duplicate entries were removed, and the remaining articles were reviewed by the first author (A.E.B.). Titles (±abstracts) were screened (Figure 1), and relevant full-text articles were reviewed. Of the eligible articles, the data extracted included the following, where it was provided: aim of intervention, duration of intervention, method of intervention delivery, intervention content, population targeted, instruments used to measure outcomes, measured outcomes, and limitations. Any discrepancies in data analysis were discussed with the principal investigator (C.J.M.). 

The selected studies were analyzed according to the logistics of intervention implementation (number and length of sessions, duration of intervention, school staff involvement), and the measured impact of each intervention. 

## 3. Results

The search strategy yielded 7222 articles (Figure 1) once duplicates were removed. After screening titles and abstracts, 7208 articles were deemed ineligible and 46 remained for full-text review. After full-text review, five published studies were eligible for analysis.

### 3.1. Overview of Studies

The five included studies (Table 1) were published over a 23-year period, with four of the five studies published in a 9-year period between 2013 and 2021. Sample sizes ranged from 71 [23] to 3135 [24] children. Experiential cookery opportunities formed the basis of the interventions, with time also given to food tasting [24,25], food gardening [24,25,26], and/or nutrition education [23,24,25,26]. Four studies [23,24,25,27] targeted children aged 7–11 years and one study [26] targeted children aged 5–12 years. Parents were actively included in the delivery of two [26,28] of the five interventions.

### 3.2. Intervention Aims and Content

The aims of the included interventions encompassed outcomes such as increasing food knowledge [24,26], improving attitudes toward cooking and plant foods [25,26,27], and enhancing cookery skills [25,26,27] and dietary outcomes [24,26]. Although aims varied across interventions, there were similarities in the content delivered to achieve these aims.

The quasi-experimental Veggiecation intervention [23] (Table 1) aimed to increase vegetable consumption and to improve children’s preferences for, and attitudes about, vegetables. The intervention consisted of four classes, delivered weekly, where two vegetables were introduced as part of each session. A short lesson to introduce each vegetable and its benefits preceded a 30 min cooking session using the vegetables of interest. Recipes required students to mix, dice, slice, blend, and mash foods in order to create dishes, such as spinach na-mul, mushroom na-mul, and cucumber salad. Parents did not receive intervention content beyond the recipe sheets students brought home after a session.

The Cooking With Kids intervention [25] (Table 1) was an RCT that aimed to expose children to fresh and affordable foods. The intervention group received three cookery sessions that involved students preparing and tasting Chinese–American fried rice with vegetables, East Indian lentils with carrot and raisin pilaf, and potatoes persillade with cabbage. This group also received three tasting sessions with four varieties each of citrus, pears, and salad greens. Parents were not reported to have received any intervention content.

The Texas Sprouts intervention [24] (Table 1) was a multi-stakeholder cluster RCT that aimed to examine the effects of a gardening, nutrition, and cooking intervention on dietary intake, obesity outcomes (weight, BMI, BMI *z*-score, BMI percentile, waist circumference, and % body fat), and blood pressure. The final curriculum for delivery consisted of 18 (11 cookery and 7 tasting) sessions that addressed nutrition concepts, such as growing and preparing fruits and vegetables, cooking healthily, making nutritious food choices, eating locally produced food, and eating healthfully in food desert neighborhoods. Sessions were specifically culturally tailored to Hispanics, a key target group of the intervention. The curriculum was also designed to be delivered outdoors; however, 34% of sessions were delivered indoors due to adverse weather conditions. This resource-intensive intervention also facilitated the development of gardens in all intervention schools, where each garden included vegetable beds, herb beds, a shed for tools and materials, a whiteboard, and seating for classes. Materials and supplies were provided to schools for garden maintenance. Parents were also offered monthly sessions on topics such as healthy shopping, importance of family meals, and increasing access to healthy foods. The intervention was implemented by full-time trained nutrition and garden educators, but key stakeholders, such as teachers, parents, community members, school staff, and students, were included on Garden Leadership Committees to support the design, build, and maintenance of gardens. 

In contrast, Jamie Oliver’s Kitchen Garden Project (JOKGP) [27] (Table 1) was broader in its aims, focusing less on specific anthropometric and diet quality changes, to instead target food enjoyment and experiences, food neophobia, and food fussiness. It was fully delivered in each school by two school staff members to pupils aged 7–9 years; the staff members had received training to deliver the intervention through the Jamie Oliver Food Foundation. Pupils prepared a variety of dishes, including mini burgers and roasted stuffed peppers, and were given recipe sheets to take home. Beyond the potential receipt of the recipe sheets provided to students, parents were not included in the intervention. 

The quasi-experimental Cookshop Program [26] (Table 1) primarily aimed to increase children’s consumption of minimally processed wholegrains and vegetables. Secondary aims included enhancing children’s preferences for, attitudes about, and knowledge of, these foods. The intervention included a lunchroom component, classroom component, and parent and community component. The four conditions of the classroom component were: cooking only, food and environment lessons (FEL) only, cooking + FEL, and no intervention. The intervention worked with school lunchrooms to make 13 particular wholegrain and vegetable foods available to children in each menu rotation cycle. These 13 foods were also the focus for classroom cooking activities, where children prepared and cooked recipes, such as spicy sweet potatoes, vegetables and three-bean chili, and salad with homemade dressing. FEL included topics such as environmentally friendly behaviors, the role of plants in health, and how people and nature cooperate in a food system. The parent and community component included hosting parent workshops and issuing a monthly “Diets and Dollars” newsletter to the households of all children involved in the intervention, 22,000 households in the local community, and another 80,000 households city-wide.

Despite differences in the content used to achieve the aims of each study, interventions generally shared a plant-based focus to the cookery sessions, an appreciation of new foods through teaching and tasting, and an understanding of where food originates.

### 3.3. Approach to Intervention Delivery

The duration of interventions varied (Table 1), ranging from four weeks to one academic year (approx. 35–40 weeks). Of the sessions delivered in each intervention, the number of sessions specifically for cookery varied from 3 to 20 sessions, with a median of 9 sessions per intervention specifically for cookery. The overall number of sessions in each intervention ranged from 4 [23] to approx. 20 [27] sessions, with a median of 18 sessions per intervention. One intervention, JOKGP [27], delivered cookery sessions exclusively, whereas the remaining interventions [23,24,25,26] allocated sessions and/or time to gardening, food tasting, and/or nutrition education alongside cookery. 

Classroom teachers played a role in delivering three [25,26,27] of the five interventions (Table 1). Perhaps due to the need to ensure fidelity to the randomized controlled design of the Texas Sprouts [24] and Cooking with Kids [25] interventions, sessions were led by persons external to school staff. The Texas Sprouts intervention [24] was delivered exclusively by persons external to the teaching staff, whereas teachers served as supporting staff on the delivery of the Cooking With Kids [25] intervention. The Veggiecation [23] intervention, although not an RCT, was led by a nutrition teacher who was supported by teacher assistants. Of the five interventions, sessions were generally 1-2 h in duration, except for Veggiecation [23] sessions, which each took 40 min of the school day. Sessions were delivered weekly or fortnightly. 

Parents were actively included in two of the five interventions [24,26] (Table 1). In the Texas Sprouts intervention [24], parents were facilitators and recipients of the intervention. A number of parents were included as members of Garden Leadership Committees, and all parents were offered 9 monthly sessions on nutrition and gardening topics, separate to the children’s sessions. These sessions were held during, and outside of, school hours, depending on the preference of the school site. Parents were encouraged to participate in sessions through incentives, such as free meals and groceries, water bottles, free childcare for children and siblings, and raffles for gift cards. Despite the encouragement to attend, only 7.1% of potential participating parents attended one or more sessions, and fewer than 1% attended 50% (*n*5) of the parent sessions. In the Cookshop Program [26], parents were also facilitators and recipients of the intervention, where one parent was assigned to each classroom in which the intervention was delivered, and all parents in the study schools received monthly newsletters on topics such as buying, storing, and preparing wholegrains and vegetables.

### 3.4. Evaluation and Outcomes

As shown in Table 2, the measured outcomes varied considerably between studies, although there was generally consistent reporting of findings on children’s relationships with fruits, vegetables, and cooking self-efficacy.

The Veggiecation intervention [23] was evaluated (Table 1), using a pre-post survey. Cronbach’s alpha for survey items ranged from 0.508 to 0.777 across the scales, indicating a mix of scales with poor to acceptable reliability. Feedback was also collected after each session to assess students’ comprehension of the session content, satisfaction with the cookery, and taste preferences for the vegetables of interest that day. No differences were detected in the baseline characteristics of participants in the intervention and control groups. After the intervention, there was a significant improvement in vegetable consumption behavior among children in the intervention group, when compared to controls (Table 2). Statistically significant improvements in self-reported scores for vegetable-related intentions, attitudes, and preferences were also evident among students in the intervention. The pre-post scores in the control group did not differ. Positive changes in vegetable-related perceptions and behaviors were reported; however, the results must be interpreted with caution due to the relatively short duration of the intervention, reliance on self-reported measures for evaluation, and lack of randomized design, long-term follow-up, and objective dietary measures.

The Cooking With Kids intervention [25] was evaluated (Table 1) using a pre-post 35-item survey. The validity and test–retest reliability of this survey was confirmed [29]. When compared to the control group, positive and statistically significant changes (Table 2) were seen with vegetable preference scores, attitudes toward food and cooking, and food and cooking self-efficacy. In particular, male students and students without previous cooking experience showed the greatest positive improvements in attitudes toward cooking. Of note, a statistically significant greater proportion of children in the intervention reported cooking at home and making food with their family at baseline. The authors of this study also acknowledged that changes in behaviors at home or dietary intake as a result of the intervention were not measured, nor were parental attitudes. Overall, this study demonstrated the potential positive impact of a relatively short and focused intervention on children’s attitude toward, and relationship with, healthful foods. 

To evaluate the Texas Sprouts intervention [24], a team of 10 researchers took anthropometric measurements over the course of one week in each school. As part of the questionnaire pack, an adapted version of the School Physical Activity and Nutrition (SPAN) dietary screener was used to obtain data on fruit, vegetable, and sugar-sweetened beverage consumption. The adapted SPAN dietary screener was deemed to have moderate-to-strong agreement for reliability and moderately reproducible validity. Questions were also asked on areas such as food and meal choice behaviors, cooking self-efficacy, and gardening. Parents provided baseline demographic information that was published [24], and data on family eating activities, household food security, and parental dietary intake were also amassed but not yet reported. No significant differences were evident in the baseline data of intervention and control groups. A statistically significant improvement in vegetable intake (Table 2) was seen at the end of the intervention, with intervention children having +0.33 portion/d, compared to a change of +0.03 portion/d in the control group. No changes in any of the other targeted outcomes were evident (Table 2). The authors suggested that this may have been the result of several factors, to include the less intense (i.e., not weekly) delivery of the intervention and the potential need to facilitate more intense dissemination initially and/or have longer follow-up to reduce markers of obesity. The difficulty of encouraging parental participation was also noted, where only 7.1% attended ≥1 of the 9 monthly sessions, and as such, the impact of parents on target outcomes is unclear. The relative lack of impactful outcomes, despite the resource intensive and inclusive design of the RCT, also called into question the long-term sustainability of the intervention.

The JOKGP [27] was evaluated using a survey that was assessed for test–retest reliability. The Cronbach’s alpha for all scales ranged from 0.64 to 0.86, indicating acceptable to good reliability. After the intervention, children in the intervention group were significantly more likely to help with cooking at home and to report liking cooking when compared to the control group; findings that were also echoed in the data provided by parents. The intervention group also had improved, but not statistically significantly improved, food fussiness scores at the end of the intervention, compared to baseline. This intervention reported positive improvements in food enjoyment and cooking experiences, but similar to the Veggiecation intervention, the results must be interpreted with caution due to the lack of randomized design and long-term follow-up. 

The Cookshop Program [26] was evaluated using age-specific questionnaires (Table 1) and visual estimates of plate waste. The questionnaires each consisted of five scales that were specifically chosen to reflect the affective, cognitive, and behavioral factors impacting fruit and vegetable consumption. Cronbach’s alpha ranged from 0.23 to 0.66 across the scales, indicating a mix of scales with poor to good reliability. Baseline scores for the scales were not reported, making it difficult to fully assess the impact of the intervention. When compared to the control group (Table 2), students in receipt of cookery classes had higher post-intervention scores for knowledge, self-efficacy in cooking, behavioral intention, and preferring the target foods of the intervention. FEL also positively impacted knowledge, but to a lesser degree than the cookery sessions. FEL had no impact on food preferences, attitudes, or self-efficacy, emphasizing the value of experiential learning. Students in receipt of cookery sessions + FEL left the least amount of the 13 target foods on their plates, with 5–9-year-olds and 9–12-year-olds leaving 79% and 74% of the target foods on their plates, respectively. Food waste among of the 13 target foods progressively increased among students receiving only cookery classes or only FEL or no intervention. Baseline % food waste was not reported in this manuscript, making it difficult to evaluate the full impact of the intervention on reducing food waste. The lack of reporting of baseline data, alongside the non-randomized study design, are notable limitations in interpreting the impact of this study. 

## 4. Discussion

It is clear from this review that experiential cooking opportunities, alone or in combination with other activities, have the potential to positively impact, at least in the short-term, aspects of the relationship children develop with food. The studies included in this review particularly demonstrated improvements in self-reported attitudes toward vegetables, fruits, and cooking, and two studies [24,26] reported small increases in vegetable intake. However, the current paucity of methodologically rigorous interventions with long-term follow-up precludes definitively quantifying the benefits of school-based experiential cookery interventions. As such, the type and dose of school-based cookery intervention needed to precipitate sustainable health changes among schoolchildren remain unknown. 

As reported in the systematic review by Charlton et al. [30], there are several characteristics associated with the content of school-based interventions that increase the likelihood of achieving successful outcomes. These include: frequent exposure to experiential activities that are accompanied by relevant theory-based lessons; multicomponent interventions; involvement of appropriate external personnel; contextually appropriate activities; and the inclusion of parents and take-home activities [30]. Of the studies included in this review, two (Texas Sprouts [24] and the Cookshop Program [26]) possessed all five of these characteristics. Despite reporting a small improvement in vegetable consumption, Texas Sprouts did not elicit the range of health benefits expected of its design and content. The Cookshop Program elicited positive changes in several self-reported measures and a small increase in vegetable intake, but similarly experienced challenges in provoking significant behavioral change in response to the intervention. 

To deliver health-related content to children, the use of themed content and teaching through play were found to be valuable, as has cross-curricular teaching [30,31]. As evident from the included studies, the combination of nutrition education, cookery, and food gardening increases the likelihood of achieving some degree of food-related behavior change. Furthermore, embedding the learning from these components into other curriculum subjects, such as science, mathematics, and geography, can further increase instances of exposure to an intervention [9,32,33]. Targeting as many facets of the environment as possible increases the potential to positively impact health knowledge and behavior [14,34].

However, to achieve this degree of intervention integration in a school environment, high levels of self-efficacy and subject knowledge among teachers are needed [35]. Staff buy-in, training, and support are essential to the effective and sustainable delivery of interventions [19], even if the implementation of the core intervention is led by personnel external to school staff. Limited time for interventions is a common barrier reported by teachers [19,20,21], particularly in light of the many other subjects that must be taught as part of primary/elementary school curricula [19]. As such, interventions that are designed to reflect existing curricula can help overcome some of the objections raised by teaching staff in relation to the logistics of implementation [19,34]. For example, teachers who participated in the JOKGP [27] reported in a qualitative study [34] that the links between the intervention and curriculum were a factor in: contextualizing the intervention for students; enabling teachers to achieve cross-curricular learning outcomes; and increasing student enjoyment of the sessions. Three of the five studies included in this review included classroom teachers in some aspect of the delivery, and this is an important element of the sustainability and longevity of an intervention in a school, as limited resources may make it difficult to rely solely on external personnel for implementation.

Parents are also part of the school environment, and as such, are considered integral to the success of interventions [19,30,36]. However, as was evident from this review, parental knowledge and behavior were actively targeted in only two [24,26] of the five studies. The Cookshop Program predominantly provided information that required no response from parents, i.e., newsletters, whereas Texas Sprouts [24] invited parents to attend and participate in classes on nutrition and gardening. Notably, only 7.1% of eligible parents attended ≥ 1 of the sessions offered as part of Texas Sprouts, despite consistent encouragement and offers of incentives [24]. There is a consensus in the literature that optimal methods of promoting parent involvement are difficult to determine [30,37], with indirect methods of involving parents (e.g., sending recipe sheets home with students) remaining common. Given that children consume up to 87% [4] of their total energy intake at home, parental involvement in, and support of, school-based culinary interventions is key to bridging healthful exposures in school with those at home [30,38,39,40].

The authors acknowledge the limitations of this review. The small number of studies that took place during school hours limits the potential to identify trends and make recommendations, and possibly highlights the challenges of securing a commitment from school staff to host such interventions as part of their curricula [19,20,21]. In addition, it is challenging to draw definitive conclusions on effectiveness when just two of the five studies were randomized and there was no long-term follow-up of outcomes in any intervention. In terms of the limitations in conducting this review, one author took the lead on identifying studies from the literature. However, the first author conferred with the principal investigator (C.J.M.) on the application of the inclusion criteria to the full-text manuscripts. The comprehensive systematic search strategy was constructed with the assistance of a medical librarian (D.M.), and the results were presented narratively to reflect the diversity of outcomes reported among the included studies.

It is clear from the studies included in this review that school-based culinary interventions have at least a small positive effect of unknown duration on the relationship children have with food. However, given the generally multi-faceted, multi-stakeholder, and resource-intensive designs of these interventions, sustainable implementation and evaluation remain a challenge. Considering that improved nutritional intake and dietary quality [12,13] are related to the development of cooking skills, and that the school environment is identified as a key setting in which to host health-related interventions [14], research is needed to further explore the potential of school-based culinary interventions to impart lifelong health-related skills. The literature will benefit from reports on RCTs with larger sample sizes and longer phases of implementation [14,30] to inform decision making. The consistent use of well-validated instruments [41] to assess health and psychosocial outcomes will also help inform the development and success of future interventions aiming to enhance the health and wellbeing of all children and their wider school communities.

## Figures and Tables

**Table 1:** Characteristics of school-based culinary interventions for 5–12-year-old children.

First Author	Bai et al. [23]	Cunningham-Sabo et al. [25]	Davis et al. [24]	Ensaff et al. [27]	Liquori et al. [26]
**Program name**	*Veggiecation*	*Cooking with Kids*	*Texas Sprouts*	*Jamie Oliver’s Kitchen Garden Project*	*Cookshop Program*
**Year**	2018	2013	2021	2016	1998
**Country**	South Korea	U.S.A.	U.S.A.	U.K.	U.S.A.
**Age range (years)**	7–8	9–10	8–11	7–9	5–12
**Total *n***	71	257	3135	338	590
**Intervention *n***	35	137	1412	164	4 class groups
**Total no. sessions**	4	6	18	NS †	20
**No. cookery sessions**	4	3	11	NS †	9
**Length of sessions**	40 min	120 min	60 min	90 min	90–120 min
**Interval**	Weekly	1–2 weeks	Approx. fortnightly	Fortnightly	Weekly
**Duration**	One month	One semester	One academic year	One academic year	One academic year
**Study design**	Quasi-experimental	RCT	Cluster RCT	Longitudinal comparative	Quasi-experimental
**Theory**	NS	NS	Social ecological-transactional	Social cognitive	Social cognitive
**Content**	10 min theory lessons 30 min cookery lessons	3 cookery sessions 3 tasting sessions	11 cookery sessions 7 tasting sessions	Cookery sessions †	9 cookery sessions 17 garden trips 10 classroom lessons
**Facilitators**	Nutrition teacher Teaching assistants	Food educator Classroom teacher Nutrition graduate	Nutrition educator Garden educator	Classroom teachers	Classroom teachers Parents University students
Included parents *	No	No	Yes	No	Yes
**Evaluation method**	Pre-post survey	35-item pre-post survey	*Pre-post data included* Demographic survey SPAN dietary screener Weight Waist circumference Body composition Blood pressure % attendance	Pre-post survey	Visual estimate of plate waste (pre-post) *K–3rd grade* 38-item survey (post) *4th–6th grade* 67-item survey (post)

NS: Not specified; RCT: Randomized Controlled Trial; SPAN: School Physical Activity and Nutrition; † Based on duration of an average academic year in the UK (approx. 40 weeks), estimate that 15–20 cookery sessions were delivered; * Parents included as facilitators and/or recipients of the intervention.

**Table 2:** Summary of key results from school-based culinary interventions for 5–12-year-old children.

**Bai et al.** [23]	Compared to the control group, the Veggiecation intervention led to the following: + Higher (*p* < 0.001) self-reported scores for behavior and intention in relation to vegetables. + Higher (*p* < 0.001) self-reported scores for attitude about, and preference for, vegetables. + Higher (*p* < 0.01) self-efficacy scores in relation to vegetables. + A total of 43.3% of parents reported trying the recipes at home.
**Cunningham-Sabo et al.** [25]	Compared to the control group, the Cooking with Kids intervention led to the following: + Higher (*p* = 0.007) vegetable preference scores. + More positive (*p* = 0.029) attitude toward food and cooking. + Positive (*p* < 0.001) change in food and cooking self-efficacy. - Higher (but not statistically significant, *p* ≤ 0.087) fruit preference scores.
**Davis et al.** [24]	Compared to the control group, the Texas Sprouts intervention led to: + Higher (*p* = 0.02, CCA) frequency of vegetable intake. - No significant change to frequency of fruit (*p* = 0.77) and SSB (*p* = 0.15) intake. - No significant change in CCA for BMI (*p* = 0.84), BMI z-score (*p* = 0.36), or BMI percentile (*p* = 0.39). - No significant change in CCA for waist circumference (*p* = 0.31) or percentage body fat (*p* = 0.47). - No significant change in CCA for systolic (*p* = 0.81) and diastolic (*p* = 0.28) blood pressure.
**Ensaff et al.** [27]	Compared to the control group, Jamie Oliver’s Kitchen Garden Project led to the following: + Higher scores (*p* = 0.004) for liking cooking. + Increased likelihood (*p* = 0.034) of helping with cooking at home. + Higher (*p* = 0.004) taste description score. + Higher (*p* = 0.02) scores from parents for children liking cooking. - No significant change (*p* = 0.493) in the food neophobia and fussiness score.
**Liquori et al.** [26]	Compared to the control group, the Cookshop Program led to the following: + Higher (*p* ≤ 0.001) mean preference scores for plant foods. + Positive (*p* ≤ 0.001) impact on knowledge. + Positive (*p* ≤ 0.05) impact on self-efficacy in cooking. + Positive (*p* ≤ 0.10) effects found for plate waste, i.e., increased consumption of targeted foods. + Higher (but not statistically significant) scores for intentions to eat plant foods. - No statistically significant change in attitudes to health, cooking, and cooperation.

BMI: Body Mass Index; CCA: Complete Cases Analyses; SBB: Sugar-Sweetened Beverages.

## Data Availability

Not applicable.
